# Differential regulation of host plant adaptive genes in *Pieris* butterflies exposed to a range of glucosinolate profiles in their host plants

**DOI:** 10.1038/s41598-019-43703-8

**Published:** 2019-05-10

**Authors:** Yu Okamura, Ai Sato, Natsumi Tsuzuki, Yuji Sawada, Masami Yokota Hirai, Hanna Heidel-Fischer, Michael Reichelt, Masashi Murakami, Heiko Vogel

**Affiliations:** 1 Community Ecology Lab., Faculty of Science, Chiba University, 263-8522 Chiba, Japan; 2 RIKEN Center for Sustainable Resource Science, 1-7-22 Suehiro-cho, Tsurumi-ku, Yokohama, Kanagawa 230-0045 Japan; 3 Department of Entomology, Max Planck Institute for Chemical Ecology, Hans-Knöll-Str. 8, 07745 Jena, Germany; 4 Leibniz Institute for Natural Product Research and Infection Biology Hans Knöll Institute (HKI), Beutenberg-Str. 11a, 07745 Jena, Germany; 5 Department of Biochemistry, Max Planck Institute for Chemical Ecology, Hans-Knöll-Str. 8, 07745 Jena, Germany

**Keywords:** Biodiversity, Molecular ecology, Coevolution

## Abstract

Specialist herbivores have often evolved highly sophisticated mechanisms to counteract defenses mediated by major plant secondary-metabolites. Plant species of the herbivore host range often display high chemical diversity and it is not well understood how specialist herbivores respond to this chemical diversity. *Pieris* larvae overcome toxic products from glucosinolate hydrolysis, the major chemical defense of their Brassicaceae hosts, by expressing nitrile-specifier proteins (NSP) in their gut. Furthermore, *Pieris* butterflies possess so-called major allergen (MA) proteins, which are multi-domain variants of a single domain major allergen (SDMA) protein expressed in the guts of Lepidopteran larvae. Here we show that *Pieris* larvae fine-tune NSP and MA gene expression depending on the glucosinolate profiles of their Brassicaceae hosts. Although the role of MA is not yet fully understood, the expression levels of NSP and MA in larvae that fed on plants whose glucosinolate composition varied was dramatically changed, whereas levels of SDMA expression remained unchanged. In addition, we found a similar regulation pattern among these genes in larvae feeding on *Arabidopsis* mutants with different glucosinolate profiles. Our results demonstrate that *Pieris* larvae appear to use different host plant adaptive genes to overcome a wide range of glucosinolate profiles in their host plants.

## Introduction

Understanding the mechanisms that define the host plant ranges of herbivores is key to understanding the coevolution of plants and herbivores. Specialist herbivores have restricted host ranges but usually possess adaptive strategies to overcome the chemical challenges (i.e. defensive secondary metabolites) of their host plants^1^. Recently, in a number of generalist and specialist herbivores, the molecular adaptive mechanisms to specific groups of secondary metabolites have been partially identified, such as cytochrome P450 in *Papilio* butterflies against furanocoumarins or UDP-glycosyltransferases from *Helicoverpa armigera* or *Helicoverpa zea* to capsaicin^1–4^. Although several previous studies have investigated the molecular adaptive mechanisms with which generalist herbivores respond to different host plants^5–8^, how specialist herbivores overcome the diversity of the secondary metabolites is not well understood. The same holds true for specialist herbivores that have been exposed to chemical variation in their host plants, since secondary metabolites are often diversified chemically within plant families and even between closely related species^9,10^.

Glucosinolates (GLSs), a group of secondary metabolites found in Brassicales, are stored in plant cells separated from specific enzymes called myrosinases^11^. In response to tissue damage in plants, GLSs come into contact with myrosinase enzymes and are hydrolyzed, forming several breakdown products^12^. Among these, the isothiocyanates (ITCs) are dominant and are toxic to numerous herbivores^11^. GLSs are divided into three major classes depending on their biosynthetic origins (aliphatic GLSs, benzylic GLSs and indolic GLSs) and are further sorted according to a variable side chain, with more than 140 different GLSs identified so far^9,13^. Notably, most of these variations seem to be present in the family Brassicaceae, with each species having a specific GLS profile^9,14^.

Although the GLS myrosinase system in Brassicales is known as an efficient defense against many herbivores^15,16^, some insects can use Brassicales plants as hosts^1,4^. Pierid butterflies use Brassicales plants by disarming GLS defense with a protein expressed in the larval gut called nitrile-specifier protein (NSP)^17^. NSP can redirect the GLS-myrosinase reaction to form less toxic nitriles rather than toxic ITCs. Since Pierid butterfly larvae are generally oligophagous and use a distinct range of Brassicales host plants^18^, each species will likely be exposed to a range of GLSs in their host plants. *Pieris* butterflies encounter a large variety of GLS profiles in their host plants because they rely on various genera in the Brassicaceae family, which seems to have the highest GLS diversity in Brassicales^14,19^.

NSP belongs to the small so-called NSP-like gene family, which also includes genes that encode major allergens (MA) and single domain major allergens (SDMA)^20^. NSP and MA have a similar genetic structure, as they both possess three replicated domains that originated from SDMA^20^. SDMA is generally found across Lepidoptera. Although its specific function is still unknown, SDMA is known to be expressed only in larval guts, suggesting this protein plays a general digestion-related role in Lepidoptera^21^. NSP and MA are specific for Pierid butterfly larvae feeding on Brassicales^20^, suggesting that both proteins may have GLS-disarming functions. The role of MA proteins in modifying the outcome of the GLS-myrosinase defense system, by which MA causes nitriles rather than ITCs to form, was confirmed in a Brassicaceae-feeding Pierid species, *Anthocharis cardamines*, which also seems to lack an NSP gene^19^. However, in general, the role of MA proteins is still unclear in most *Pieris* species, especially those which encode both NSP and MA^19^. Given that in at least one *Pieris* species, not only NSP but also MA have been shown to have GLS-disarming functions, the presence of both genes in some Pieridae could be a molecular adaptive mechanism for overcoming diverse GLSs in the species’ host plants.

**Figure 1:**
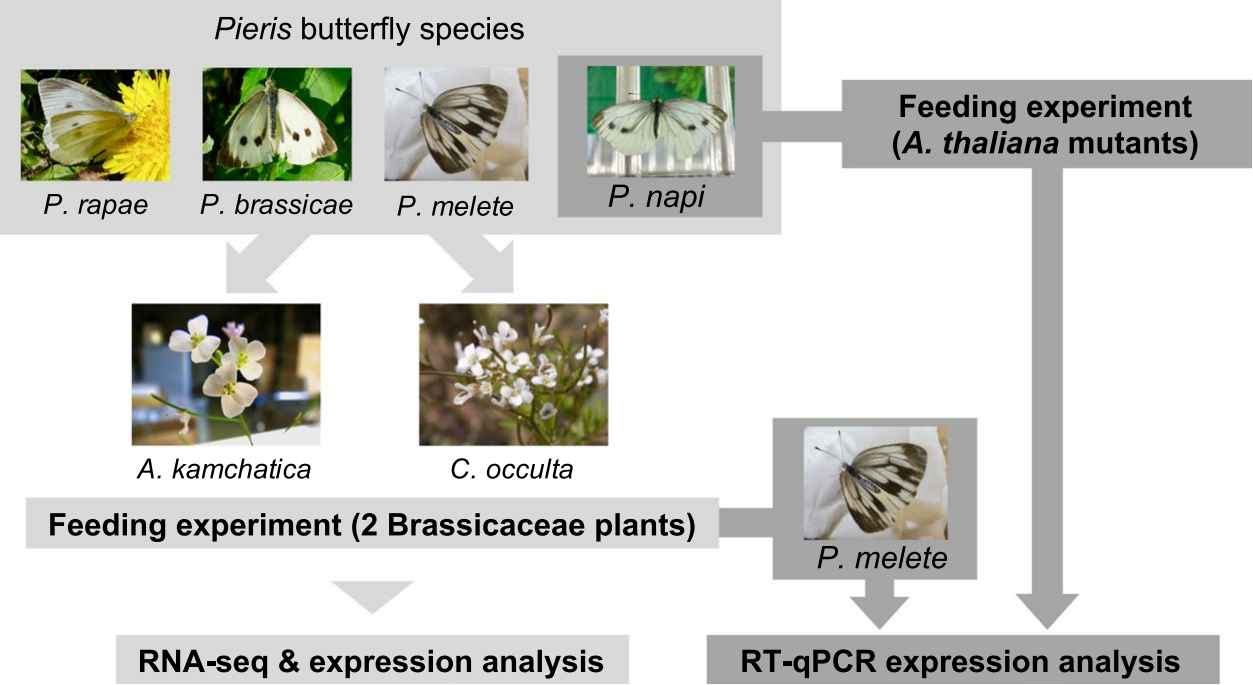
Experimental design of this study. We focused on four *Pieris* butterflies, and conducted feeding experiments with two Brassicaceae plants in order to see gene expression patterns of NSP-like gene family members to different host plants with distinct GLS profiles by RNA-seq. We confirmed the result of RNA-seq based expression analysis by RT-qPCR using *P*. *melete* as a representative with more replicates. We also conducted feeding experiments with *Arabidopsis thaliana* mutants which differ in their GLS profiles to get gene expression data in more controlled chemical background. For this mutant experiment, we used *Pieris napi* as a representative.

Here, we focus on members of the NSP-like gene family and their regulation patterns in Brassicaceae-feeding *Pieris* larvae exposed to a range of GLS profiles, in order to find out how *Pieris* butterflies respond and adapt to diverse GLSs in their host plants. We conducted feeding experiments using four Japanese *Pieris* species (*Pieris napi*, *P*. *melete*, *P*. *rapae* and *P*. *brassicae*) and two Brassicaceae plants (*Arabidopsis kamchatica* and *Cardamine occulta*, both of which have diverse GLS profiles)^9,13,22,23^. The four closely related *Pieris* species are known to have different host ranges. *P*. *napi* and *P*. *melete* mainly use wild Brassicaceae plants such as *Arabis* or *Cardamine* in Japan^24,25^, while *P*. *rapae* and *P*. *brassicae* are known pests of *Brassica* crops (Table 1)^26–28^. NSP sequences have been identified only in *P*. *rapae* and *P*. *brassicae* in this genus^19,20,29^. We first identified NSP, MA and SDMA sequences in all of the tested *Pieris* species, combining high throughput RNA sequencing (RNA-seq) of larval samples from our plant-feeding experiments and *de novo* transcriptome assemblies. We subsequently measured and compared the expression levels of all of the identified NSP, MA and SDMA genes in larvae that fed on two host plants using the RNA-seq data. To confirm the expression levels of the NSP-like gene family observed in our RNA-seq data with a larger number of replicates, we also conducted real-time quantitative PCR (RT-qPCR) on *P*. *melete*, considering it to be a representative species and targeting all members of the NSP-like gene family (Fig. 1).

**Table 1:** Four *Pieris* butterflies and their main host plant genera in Japan.

Species	Major Brassicaceae host	Reference
***Pieris melete***	*Arabis* *Cardamine* *Orychophragmus* *Rorippa*	Ohsaki & Sato (1994) Kitahara (2016) unpublished data
***Pieris napi***	*Arabidopsis* *Arabis* *Cardamine* *Rorippa*	Ohsaki & Sato (1994) Kitahara (2016) unpublished data
***Pieris rapae***	*Brassica* *Rhaphanus* *Rorippa*	Ohsaki & Sato (1994) unpublished data
***Pieris brassicae***	*Armoracia* *Brassica*	Ueno (1996)

Since GLS profiles are specific for each Brassicaceae plant species^9,13^, we expected to find that *Pieris* larvae feeding on the two Brassicaceae plants regulate NSP-like genes in distinct ways. However, since some plant species also differ in their general chemical profiles – excluding GLSs–in several ways, this chemical difference could also affect the regulation of genes in larvae. To obtain feeding and gene expression data using a more controlled background, we also conducted feeding experiments with *Arabidopsis thaliana* mutants, which differ in their GLS profiles but share the same chemical background. We prepared wild-type lines (Col-0) and three mutant lines of *A*. *thaliana* which have different GLS profiles–namely, MAM1 (which lacks chain elongation genes for the C3/C4 chain length variation of Met-derived GLS), MAM3 (which lacks Met chain elongation gene and has no long-chain aliphatic GLS), and quad-GLS (the quadruple mutant *myb28myb29cyp79B2cyp79B3* which has no detectable GLS)^30–32^–and used *P*. *napi* as a representative species for this feeding assay. Combining these two approaches, we were able to observe how *Pieris* butterflies regulate NSP-like gene family members in response to a broad range of GLS defenses in their host plants (Fig. 1).

## Results

### RNA-seq identified NSP-like gene family sequences of the four *Pieris* butterflies

**Figure 2:**
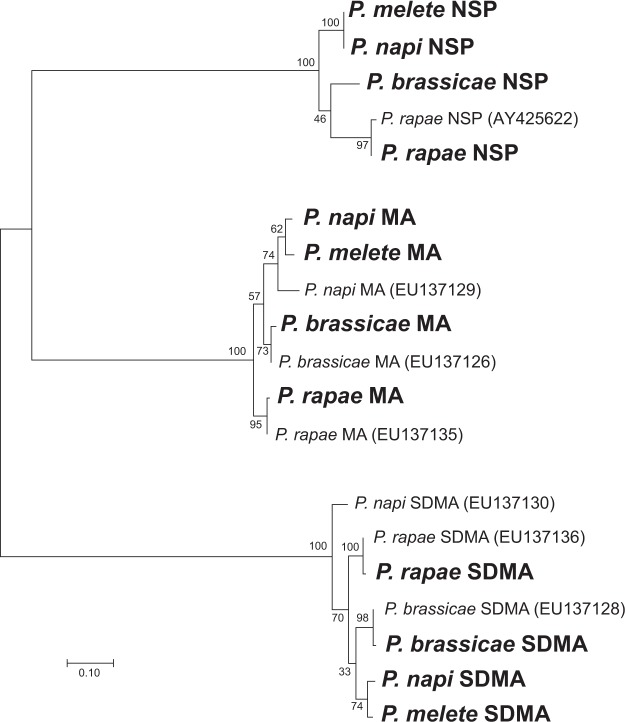
Sequence similarities of NSP-like gene family members in *Pieris* spp. acquired from RNA-seq analysis. The tree is based on amino acid sequences and was generated using the Maximum Likelihood Method. Values shown at each node are Bootstrap values. Reference sequences from GenBank are provided with accession numbers. According to the RNA-seq data analysis, all four *Pieris* species encode for NSP, MA and SDMA proteins.

We obtained 32–40 million Illumina100 bp paired-end reads from each of the four *Pieris* larval RNA samples. *De novo* transcriptome assemblies using Trinity resulted in 64,279; 62,054; 59,327; and 53,004 contigs, and in N50 values of 2,048 bp; 2,132 bp; 2,060 bp; and 2,594 bp for *P*. *napi*, *P*. *melete*, *P*. *rapae* and *P*. *brassicae*, respectively. We identified NSP, MA and SDMA sequences from all four *Pieris* butterfly species with reference sequences (Fig. 2). The newly acquired NSP sequences of *P*. *napi* and *P*. *melete* have 86% and 84% amino acid sequence identity, respectively, with NSP from *P*. *rapae*. MA proteins also showed high identity (89% each), and SDMA proteins showed slightly higher sequence identity (92% each) to *P*. *rapae*.

### NSP and MA are differentially expressed in response to wild Brassicaceae plants

**Figure 3:**
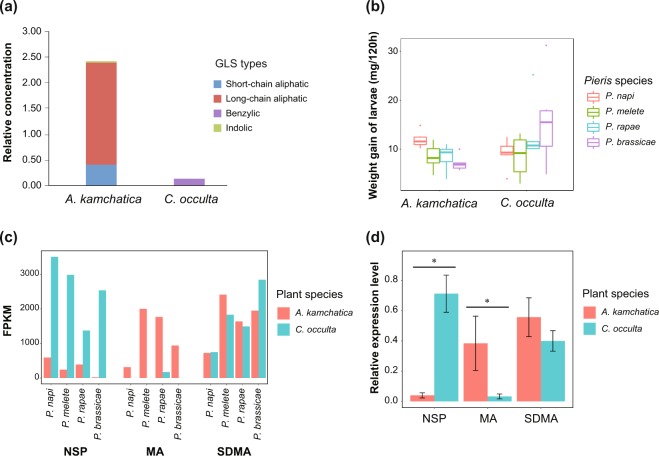
(**a**) GLS profiles of *Arabidopsis kamchatica* and *Cardamine occulta* measured by UPLC-TQMS. *Arabidopsis kamchatica* had high aliphatic GLS concentration and indolic GLS, whereas *C*. *occulta* had high concentration of benzylic GLS. (**b**) Larval growth of the four *Pieris* butterflies fed on the two wild Brassicaceae plant species used in the feeding experiment. All four *Pieris* species fed on both plant species, although not significant growth level differences were observed among the four species (pairwise *t* test with FDR adjustment, *P* > 0.05). (**c**) Relative gene expression levels of NSP-like gene family members in the four *Pieris* butterflies fed on *Arabidopsis kamchatica* and *Cardamine occulta* from digital analyses based on RNA-seq expression levels. NSP and MA showed differential expression levels in larvae fed on the two different host plant species, while SDMA did not. NSP was expressed more highly in larvae fed on *C*. *occulta*, whereas MA was expressed more highly in *A*. *kamchatica*-feeding larvae. (**d**) Relative gene expression levels (2^−ΔCt^) of NSP-like gene family members in *Pieris melete* larvae compared to larvae feeding on *Cardamine occulta* and *Arabidopsis kamchatica* analyzed by RT-qPCR. “*” show statistical significance based on the Mann–Whitney *U* test (*P* ≤ 0.05). Significant differences: NSP (*P* = 0.05), MA (*P* = 0.05), SDMA (*P* = 0.90).

GLS profiles of *Arabidopsis kamchatica* and *Cardamine occulta*, host plants of *Pieris* butterflies in Japan, were significantly different according to the UPLC-TQMS analysis. In total, we detected 25 types of GLSs in the two tested plant species and sorted these into the three major GLS chemical classes to observe their profile differences (Table S1). *A*. *kamchatica* had high amounts of aliphatic and indolic GLSs, whereas *C*. *occulta* contained benzylic GLSs, which were much lower in *A*. *kamchatica* (Fig. 3a, Table S1). Although none of the four larvae species feeding on the two plant species showed any sign of stopping feeding or of poisoning, we observed that *P*. *rapae* and *P*. *brassicae* appeared to grow better on *C*. *occulta* than on *A*. *kamchatica*. However, this difference was not statistically significant (*P* = 0.12 and 0.064, Fig. 3b).

We acquired expression levels of the NSP-like gene family from all four species of *Pieris* larvae feeding on the two Brassicaceae plants by analyzing digital expression based on a single RNA-seq data for each treatment (Fig. 3c). Compared to the observed expression levels of NSP in larvae fed with *A*. *kamchatica*, the levels increased when larvae fed on *C*. *occulta*, while an inverse gene expression pattern was observed for MA. MA was highly expressed in larvae that fed on *A*. *kamchatica*, and less expressed in larvae that fed on *C*. *occulta*. This inverse trend was observed in all four *Pieris* species, whereas expression levels of SDMA (which is not involved in disarming GLS) were similar between the larvae fed with the two Brassicaceae plants in all four *Pieris* species (Fig. 3c). The result of our *P*. *melete* larval RT-qPCR experiments also mirrored the same gene regulation pattern of NSP-like gene family members, supporting our RNA-seq results. Expression levels of NSP and MA differed significantly depending on the plant species on which the larvae fed (NSP and MA: *P* = 0.05, Mann–Whitney *U* test), whereas levels of SDMA did not (SDMA: *P* = 0.90, Mann–Whitney *U* test, Fig. 3d).

### NSP and MA are differentially regulated in *P*. *napi* feeding on *A*. *thaliana* mutants with different GLS profiles

**Figure 4:**
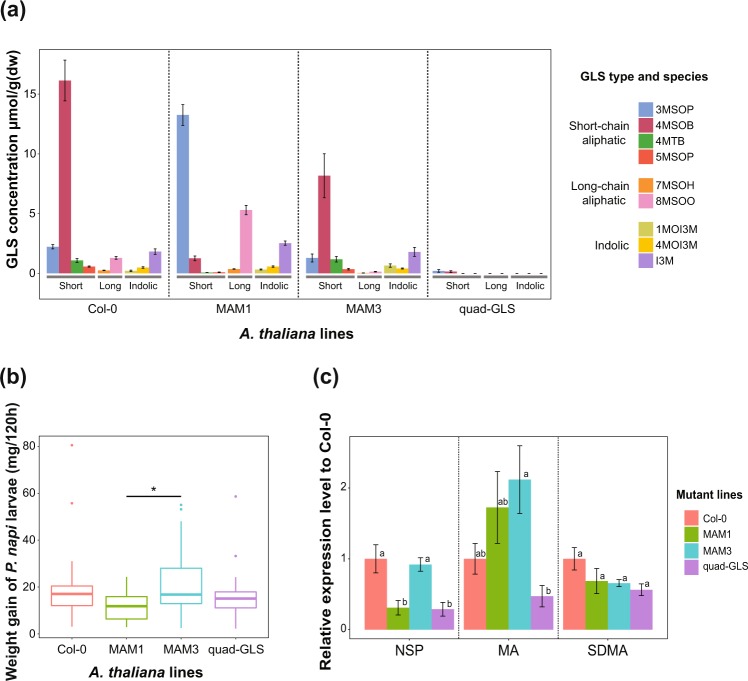
(**a**) GLS profiles of *Arabidopsis thaliana* mutants measured by LC-UV (*n* = 5). MAM1 showed lower 4MSOB concentration but accumulated higher 3MSOP and 8MSOO than Col-0. MAM3 has a lower long-chain aliphatic GLS concentration, and quad-GLS has no or quite low levels of GLSs. (**b**) Growth rates of *P*. *napi* that fed on the four *Arabidopsis thaliana* mutant lines. “*” shows significance based on the FDR-adjusted pairwise *t* test (*P* ≤ 0.05). Significant differences: MAM1 vs. MAM3 (*P* = 0.018). (**c**) Relative expression levels of NSP-like gene family members of *P*. *napi* against the four *Arabidopsis thaliana* mutants. Expression level was normalized based on Col-0 for each gene. Different letters on each box show significance (pairwise *t* test with FDR adjustment, *P* ≤ 0.05).

We identified 9 types of GLSs from the four tested *A*. *thaliana* lines (Col-0, MAM1, MAM3 and quad-GLS) by LC-UV, confirming previously described GLS profiles for all the mutant lines (Fig. 4a)^30,31,33,34^. Col-0 had higher levels of short-chain aliphatic GLSs, and we confirmed that the quad-GLS mutant had no detectable GLSs. In the MAM1 mutant, we detected a higher amount of 3-(Methylsulfinyl)propyl GLS (3MSOP) and 8-(Methylsulfinyl)octyl (8MSOO) but less 4-(Methylsulfinyl)butyl GLS (4MSOB) compared to in the wild type (Col-0)^30^. MAM3 lacked long-chain aliphatic GLSs (8MSOO or 7-(Methylsulfinyl)heptyl (7MSOH) GLS), as described previously^31^. The feeding assay performed with *P*. *napi* and the four *A*. *thaliana* mutant lines showed that *P*. *napi* larvae that fed on MAM1 grew more slowly than did those feeding on MAM3 (*P* = 0.018, FDR-adjusted pairwise *t* test) (Fig. 4b).

Regarding the expression patterns of NSP-like gene family members in *P*. *napi*, gene regulation differed significantly for NSP and MA but not SDMA in larvae as a response to the mutant lines they fed on (Fig. 4c). In the quad-GLS mutant, which does not contain GLS, NSP and MA were down-regulated in larvae; these were not down-regulated in larvae that fed on wild-type (Col-0) lines. In MAM1, which had higher levels of 3MSOP but lower levels of 4MSOB, we observed NSP in larvae to be significantly down-regulated compared to larvae that fed on Col-0 (wild type); in contrast, MA did not show this trend and had higher expression levels than in the larvae that fed on wild-type lines. When larvae fed on MAM3 lacking long-chain aliphatic GLS, NSP was expressed at levels similar to those found in larvae that fed on Col-0 but MA expression was highest and comparable to MAM1 mutant plant-feeding larvae.

## Discussion

In this study, we conducted feeding experiments combined with gene expression analysis using four *Pieris* butterflies raised on two Brassicaceae plants and four *A*. *thaliana* mutant lines. We aimed to reveal if and how NSP-like gene family members of *Pieris* butterflies respond to a broad range of GLS profiles. We found that the gene expression levels of NSP and MA in *Pieris* larvae responded to the presence of GLSs and to GLS profile differences in the plants they fed on, but levels of SDMA remained the same. In addition, gene regulation differed between NSP and MA in response to GLS profiles in host plants; surprisingly, the two members of the NSP-like gene family actually showed inverse expression patterns to the plant species. The results suggest that both NSP and MA are involved in disarming plants’ defense systems by targeting different GLSs, and both of these proteins are the result of an adaptive mechanism *Pieris* employs to overcome a wide range of GLS profiles in their host plants.

RNA-seq based gene expression analysis showed that NSP was more highly expressed when larvae fed on *C*. *occulta*, which had more benzylic GLSs compared to *A*. *kamchatica;* in contrast, MA was highly expressed in larvae that fed on *A*. *kamchatica*, which had more aliphatic and indolic GLSs (Fig. 3a,c). Unlike NSP and MA, SDMA was expressed at a similar level, regardless of which plant the larvae fed on (Fig. 3c). These expression patterns of NSP-like gene family members were observed in all four *Pieris* species (Fig. 3c), and this pattern was also confirmed with more replicates by RT-qPCR analysis in *P*. *melete* (Fig. 3d). Both *P*. *napi* and *P*. *melete* are known to be able to use both of these Brassicaceae species as hosts in the field^24,25^; therefore, these expression patterns cannot be dismissed as a response to host or non-host plant differences. Furthermore, the larval growth rate of the four species showed no correlation with the expression patterns of NSP and MA, suggesting that the observed pattern was not the result of larval performance differences in the two plants (Fig. 3b). The involvement of NSP in disarming GLSs has been shown earlier; however, our results suggest that both NSP and MA are involved in functions related to disarming GLS, since these two genes responded to two host plants with dissimilar GLS profiles. Unlike Pierid-specific NSP or MA, SDMA, which can be found across Lepidoptera, did not seem to respond to GLS profile differences, suggesting SDMA is not involved in GLS-specific detoxification or in processes redirecting the breakdown products (Fig. 3c,d). Interestingly, not only did NSP and MA respond to different host plants, but they also showed inverse expression patterns in larvae feeding on the two plants (Fig. 3c,d). NSP seems to respond to benzylic GLSs, whereas MA appears to respond to aliphatic and indolic GLS. However, since we used different Brassicaceae species in this feeding experiment, it can be assumed that non-GLS based chemical differences between the two plant species exist. Thus, these differences might also affect the regulation of larval NSP and MA gene expression.

We used *A*. *thaliana* GLS mutants (wildtype Col-0; MAM1 and MAM3 with different aliphatic GLS compositions compared to Col-0, and quad-GLS with no detectable GLS) in a feeding experiment with *P*. *napi* larvae. We found that NSP and MA but not SDMA was down-regulated in larvae that fed on quad-GLS mutants; as these mutants lack GLSs, the expression of NSP and MA must be triggered by the presence of GLSs, supporting our hypothesis that in *Pieris* larvae, both genes have GLS-disarming roles. Our results are supported by a recent study that also reported a down-regulation of NSP in *P*. *brassicae* when larvae fed on an *A*. *thaliana* mutant lacking GLSs^6^. These results suggest that there could be costs associated with high level expression of NSP and MA proteins in the larval gut. Therefore, the observed context-specific down-regulation of either NSP or MA, respectively, could be observed in larvae that feed on plants with overall lower GLS amounts, such as certain Brassicaceae crop plants. Furthermore, mRNA levels of NSP and MA also responded differently to GLS profiles. We observed that NSP was down-regulated in larvae that fed on MAM1 plants compared to those that fed on Col-0 plants (Fig. 4c). The MAM1 mutant has a different aliphatic GLS profile (especially 4MSOB) than Col-0; therefore, the level of NSP might vary in response to a type of aliphatic GLS. MA was only down-regulated in larvae that fed on quad-GLS mutants with no GLSs (Fig. 4c). Since MA was not down-regulated in larvae fed on MAM1 and MAM3 plants (which have different aliphatic GLS profiles), MA might not respond to aliphatic GLSs. Therefore, the observed down-regulation of MA in larvae fed on quad-GLS plants might be caused by a lack of indolic GLS.

When comparing the NSP and MA gene expression profile between the two feeding experiments (with Brassicaceae plant species and with *A*. *thaliana* mutants), we found discordant expression patterns only in NSP. While the level of NSP did not respond to the presence of aliphatic GLS in the two plant species assays (Fig. 3a,c), it did respond to differences in the aliphatic GLS profiles in *A*. *thaliana* mutants (MAM1 and MAM3). Although the expression response of NSP to aliphatic GLSs seems contradictory, we lack the entire benzylic GLS, which is abundant in *C*. *occulta*, in our *A*. *thaliana* mutant assay, and so cannot state definitively what such a contradiction means. However, it may be that benzylic GLSs are a major target of NSP and so able to trigger its expression, resulting in the different patterns of NSP expression observed in the two feeding experiments. Therefore, further analyses are necessary, especially those that focus on larval responses to benzylic GLSs.

In both feeding assays, the expression profile of MA was similar. The expression of MA was elevated in larvae that fed on *A*. *kamchatica*, which had more indolic GLS than did *C*. *occulta* (Fig. 3c,d), as well as in larvae that fed on mutants which have indolic GLSs. If, as mentioned above, the observed down-regulation of MA in quad-GLS *A*. *thaliana* mutants was caused by a lack of indolic GLS, MA regulation could be a result of the same GLS trigger. Although the responses of NSP and MA should be tested in specific and controlled experiments, our results suggest that NSP and MA might share GLS-disarming functions that are related but not identical in the many species of *Pieris* butterflies.

The regulation of detoxification-related gene expression in herbivores fed on different host plants have been compared in many studies^5–8^. In most of these, the authors focused mainly on differential gene expression in generalist herbivores as a result of dietary metabolites or on host plant family differences to understand the molecular mechanisms which enable a wider host plant range than in specialist herbivores. However, larval responses to gene regulation in different types of host plants in specialist herbivores have not been well tested^6^. Here we show that even specialist herbivores may fine-tune adaptive gene expression in response to variations in a class of host plants’ major chemical defenses, the GLSs.

Our study demonstrates that NSP and MA, which are members of an important gene family involved in host plant adaptation in Pieridae, are induced by the presence of GLSs and differentially expressed in larvae that were fed on plants that have different GLS profiles. In addition, we also found that the regulation patterns of NSP and MA were mostly conserved in the four *Pieris* butterfly species used in this study. Although the function of MA is still unclear, our results strongly support the idea that not only NSP but also MA is involved in the molecular adaptation mechanisms relied on by *Pieris* butterflies to overcome the GLS defense system in their host plants. This hypothesis is also supported by the fact that *A*. *cardamines*, which seems to lack NSP and has only MA, can use Brassicaceae plants as hosts^19,35^. Furthermore, our results also indicate that NSP and MA have different functions. Although the functional difference between the two genes still needs to be confirmed biochemically, our results suggest that a dynamic gene family has enabled *Pieris* butterflies to overcome the diversity of GLSs and radiate widely, becoming one of the most successful herbivore groups that feed on Brassicales plants. Further understanding the relationships between gene evolution and the function of the NSP-like gene family and the host plant spectrum of the Pieridae can therefore help to shed light on the molecular mechanisms that mediate the coevolutionary arms race between plants and herbivores.

## Materials and Methods

### Feeding experiments using two wild Brassicaceae plant species with GLS analysis

We conducted feeding experiments using four closely related *Pieris* butterfly species (*P*. *napi*, *P*. *melete*, *P*. *rapae*, and *P*. *brassicae*) and two Brassicaceae plants from different genera (*Arabidopsis kamchatica* and *Cardamine occulta*). We collected female egg-laying butterflies from three out of the four *Pieris* species from the wild population in Hokkaido (*P*. *napi*, *P*. *rapae*) and Chiba (*P*. *melete*), Japan. For *P*. *brassicae*, we collected final instar larvae in Hokkaido (Japan), reared them to adults and mated them by hand-pairing to get fertilized females. We placed the fertilized butterflies in chambers with their host plants (*Cardamine leucantha* for *P*. *napi* and *melete*, *Brassica oleracea* var. *capitata* for *P*. *rapae* and *brassicae*) under high-intensity light conditions. Acquired eggs were incubated at 25 °C and neonates were used for feeding experiments immediately after hatching. We collected seeds from the two species of Brassicaceae plants from the wild population. These seeds were watered, and germinated seeds were transplanted to vermiculite soil. We watered plants once a week with optimally diluted Hyponex solution (N:P:K = 6:10:5; Hyponex, Osaka, Japan). We reared the plants for 2 months under these conditions: 25 °C, with 60% relative humidity and L16:D8. Next, for the feeding experiments, 3 neonates were applied to one plant using a soft-haired brush. We replicated this set twice for each *Pieris* species and harvested 6 individuals in total from each plant species after 120 hours of feeding. After the harvested larvae were individually weighed (within 0.1 mg), they were flash-frozen in liquid nitrogen immediately and stored at −80 °C until RNA extraction. We conducted FDR-adjusted pairwise *t* tests to identify statistically significant differences in larval growth among the treatments for each species.

We used these plants not only for the feeding experiments but also for GLS profile analyses. We harvested leaves from three undamaged individual plants and froze them with liquid nitrogen. Leaves were freeze-dried and ground with metal beads. An aliquot of each powdered sample was pooled for each species and analyzed three times by tandem quadrupole mass spectrometry (TQMS) coupled with ultra-performance liquid chromatography (UPLC)^36,37^. We extracted peaks that showed >30 signal/noise ratios as detected peaks and identified GLSs following Sawada *et al*. (2009) and Sawada *et al*. (2017)^36,37^. The relative concentrations of each GLS among samples were calculated by comparing the peak area with the internal standard (10-camphorsulfonic acid). Detected GLSs were sorted into major GLS chemical classes: short-chain aliphatic (-C5), long-chain aliphatic (C6–8), benzylic and indolic GLS.

### RNA extraction, RNA-seq, *de novo* assembly, NSP-like gene family sequence identification and gene expression level analysis

For each butterfly species, we selected one representative larva from the two treatments (two plant species). We chose 8 larvae for RNA sequencing (larvae of 4 *Pieris* species fed on 2 different plant species each) in total. We extracted RNA with RNeasy Mini Kit (QIAGEN). Extracted RNA samples were quality checked with an Agilent 2100 Bioanalyzer, and all samples were confirmed to have high-quality total RNA. The library for RNA-seq was prepared by Sure Select Strand-Specific RNA Library Preparation Kit for Illumina Multiplexed Sequencing. We sequenced the samples individually on a HiSeq. 1500 (100 bp paired-end read technology). Acquired reads were trimmed by trimmomatic software with the following options (LEADING:10 TRAILING:10 SLIDINGWINDOW:4:20 MINLEN:40–normalize_reads)^38^. For *de novo* assembly, we pooled all of the trimmed reads from the same species. We conducted *de novo* assembly with Trinity ver. 2.0.6 for each species^39^. For identifying NSP-like gene family sequences, we used tblastn with setting the assembled contigs (backbone) as databases and NSP-like gene family protein sequences from *P*. *rapae* as queries (GenBank accession number AAR84202, ABY88945, ABY88946), and the e-value threshold was set as 1.0e-4^40^. We extracted hit contigs for each query from each species, aligned and trimmed with MEGA6 to reference sequences of NSP-like gene family members from *Pieris* species stored in GenBank^41^. We made a ML molecular phylogeny of acquired sequences with reference sequences to confirm our annotation (in amino acid level). To measure the relative expression level of each extracted gene, we excluded redundant isoforms of NSP-like gene family members observed in assembled contig backbones and replaced them with trimmed representative sequences. Relative expression levels of each gene were estimated by mapping trimmed reads on an assembled backbone by RSEM^42^. Fragments per kilobase of exon per million reads mapped (FPKMs) were used as a relative expression level for each gene.

### Gene expression analysis by qPCR

We also conducted RT-qPCR to confirm the gene expression levels of NSP-like gene family members. We chose *P*. *melete* as a representative species, and 3 larvae from each treatment were used for RT-qPCR. We designed primers for RT-qPCR analysis with the following Primer3Plus settings: product size = 70–180 bp, Tm = 59–61 °C, GC% = 40–60%, Max Poly-base = 3 for members of NSP-like gene family^43^. We also designed primers for *EF1α*, which is frequently used as a housekeeping reference gene in insects for qPCR^6^. Designed primers are listed in Table S2. We extracted RNA as described above, and after confirming the quality of RNA by Agilent 2100 Bioanalyzer, we digested gDNA from each extracted RNA sample using TURBO DNA-free Kit (QIAGEN). We synthesized cDNA with Prime Script RT reagent Kit with gDNA Eraser (Perfect Real Time) (TAKARA) after RNA purification by RNA Clean & Concentrator kit (ZYMO research). We ran RT-qPCR reactions with a CFX Connect Real-Time PCR Detection System (BIO-RAD) using SYBR Premix Ex Taq (Tli RNase H Plus) with two technical replicates for each sample. We verified specific amplification by performing a melting curve analysis from 65 °C to 95 °C. We calculated relative gene expression levels by the ΔCT method normalized by *EF1α*^44^. We conducted one tailed Mann–Whitney *U* test to see expression level differences between the treatments follow the trend we found in RNA-seq based expression analysis with software Rstudio ver. 1.0.136^45^. Raw qPCR data are available in Table S3.

### Feeding experiments using *Arabidopsis thaliana* mutants with different GLS backgrounds

We prepared one wild-type (Col-0) and three mutant lines of *Arabidopsis thaliana* which have different GLS profiles (MAM1, MAM3, quad-GLS). We grew these four lines under short day conditions (25 °C, 8L16D, 60% humidity), and used them for feeding experiments 5 weeks after germination. In this experiment, we used *P*. *napi* as a representative species. We collected *P*. *napi* larvae in Fukushima, Japan, and reared them to adults. Adults were paired by hand, and acquired neonates were used for the feeding assay. We followed the same protocol as we used for the feeding experiments with the two wild Brassicaceae plants described above. We applied 5 larvae to each mutant individual and replicated this set 4 times (*n* = 20). We harvested larvae after 120 h feeding and weighed them. We conducted FDR-adjusted pairwise *t* tests to identify statistically significant differences in larval growth among the treatments. 5 larvae from each treatment were randomly chosen and dissected for further expression level analysis. Mid-gut samples were flash-frozen and stored at −80 °C until RNA extraction. RNA was extracted with innuPREP RNA Mini Kit (Analytik Jena, Germany). We conducted RT-qPCR as described above to measure the expression levels of the NSP-like gene family. We conducted FDR-adjusted pairwise *t* tests to identify statistically significant differences in gene expression levels among the treatments. Raw qPCR data are available in Table S4.

### GLS analysis of *A*. *thaliana* mutant lines

We harvested entire rosettes of 5 individuals from each *A*. *thaliana* mutant line and froze them with liquid nitrogen. The samples were freeze-dried and ground by metal beads in a shaker. 10 mg of grounded leaf powder was used for chemical analysis. We added 80% of methanol with 50 µM of 4-hydroxybenzyl GLS (Sinalbin), which is absent in *A*. *thaliana*, to each mix as an internal standard. After 5 minutes of incubation with 230 rpm of shaking, we spun down the samples with 130,000 rpm for 10 minutes. We added the supernatant to filters conditioned with DEAE sephadex A-25. We washed the filter columns once with 500 µl of 80% MeOH and twice with 1 ml of water. After a final washing step with 1 ml of MES buffer pH5.2, we added 30 µl sulfate to convert GLS into desulfo GLS and incubated each sample overnight at room temperature. We eluted each column with 0.5 ml water and analyzed each using HPLC-UV with a reverse-phase C-18 column (Nucleodur Sphinx RP, 250 mm × 4.6 mm, 5 μm, Machrey-Nagel, Düren, Germany). Desulfo GLSs were identified based on the retention time and UV spectra with known standard libraries^46^.

## Supplementary information

Supplementary information

## Data Availability

The RNA-seq short read data have been deposited in the EBI short read archive (SRA) with the following sample accession numbers: ERX2829492-ERX2829499. The complete study can also be accessed directly using the following URL: http://www.ebi.ac.uk/ena/data/view/PRJEB29048.
