# Supplementary material for: Differential regulation of host plant adaptive genes in Pieris butterflies exposed to a range of glucosinolate profiles in their host plants
Source: Sci Rep. 2019 May 10;9:7256. doi: 10.1038/s41598-019-43703-8 (PMC6510735; doi:10.1038/s41598-019-43703-8)
Supplement: Supplementary file 1 — Supplementary information [file 41598_2019_43703_MOESM1_ESM.pdf]

Supplementary information

Differential regulation of host plant adaptive genes in *Pieris* butterflies exposed to a range of glucosinolate profiles in their host plants

Yu Okamura*[1,3], Ai Sato[1], Natsumi Tsuzuki[1], Yuji Sawada[2], Masami Yokota Hirai[2], Hanna Heidel-Fischer[3,4], Michael Reichelt[5], Masashi Murakami[1], Heiko Vogel[3]

1 Community Ecology Lab., Faculty of Science, Chiba University, 263-8522, Chiba, Japan
2 RIKEN Center for Sustainable Resource Science, 1-7-22 Suehiro-cho, Tsurumi-ku, Yokohama, Kanagawa 230-0045, Japan
3 Department of Entomology, Max Planck Institute for Chemical Ecology, Hans-Knöll-Str. 8, 07745, Jena, Germany
4 Leibniz Institute for Natural Product Research and Infection Biology
Hans Knöll Institute (HKI), Beutenbergstraße 11a, 07745 Jena, Germany
5 Department of Biochemistry, Max Planck Institute for Chemical Ecology, Hans-Knöll-Str. 8, 07745, Jena, Germany

Table S1 Detected mean GLS relative concentration (peak area/IS area) from two Brassicaceae plants (*n* = 3).

GLS	GLS Class	*A. kamchatica*	sd	*C. occulta*	sd
B3E	aliphatic	0.00548	0.00073	n.d.	
4MB3E	aliphatic	0.00001	0.00001	n.d.	
2P	aliphatic	0.11112	0.01257	0.00676	0.00074
3MSOP	aliphatic	0.11458	0.00264	n.d.	
4MSOB	aliphatic	0.00600	0.00149	n.d.	
5MSOP	aliphatic	0.07528	0.00089	n.d.	
6MSOH	aliphatic	3.09546	0.24712	0.00081	0.00061
7MSOH	aliphatic	6.15111	0.49682	0.00021	0.00022
8MSOO	aliphatic	0.17269	0.01765	n.d.	
3MTP	aliphatic	0.02138	0.00337	n.d.	
4MTB	aliphatic	0.00158	0.00189	0.00012	0.00022
5MTP	aliphatic	0.00607	0.00168	n.d.	
6MTH	aliphatic	0.12837	0.01493	<0.00001	<0.00001
7MTH	aliphatic	0.16384	0.02104	0.00001	0.00002
8MTO	aliphatic	0.00123	0.00075	n.d.	
3OHP	aliphatic	3.85571	0.13843	n.d.	
4OHB	aliphatic	0.00228	0.00129	n.d.	
3BZOP	aliphatic	0.00003	0.00002	n.d.	
4BZOB	aliphatic	0.00001	0.00002	n.d.	
Benzyl	benzylic	0.00088	0.00034	0.46314	0.00633
PE	benzylic	0.00017	0.00030	0.02360	0.00938
S2H2P	benzylic	0.00003	0.00005	0.00026	0.00017
I3M	indolic	0.11326	0.01255	0.07879	0.00908
1MOI3M	indolic	n.d.		0.00004	0.00007
4MOI3M	indolic	0.22289	0.01655	0.00078	0.00038

GLS abbreviations are as follows: B3E: But-3-enyl, 4MB3E: 4-(Methylsulfinyl)but-3-enyl, 2P: 2-propenyl, 3MSOP: 3-(Methylsulfinyl)propyl, 4MSOB: 4-(Methylsulfinyl)butyl, 5MSOP: 5-(Methylsulfinyl)pentyl, 6MSOH: 6-(Methylsulfinyl)hexyl, 7MSOH: 7-(Methylsulfinyl)heptyl, 8MSOO: 8-

(Methylsulfinyl)octyl, 3MTP: 3-(Methylthio)propyl, 4MTB: 4-(Methylthio)butyl, 5MTP: 5-(Methylthio)pentyl, 6MTH: 6-(Methylthio)hexyl, 7MTH: 7-(Methylthio)heptyl, 8MTO: 8-(Methylthio)octyl, 3OHP: 3-Hydroxypropyl, 4OHB: 4-Hydroxybutyl, 3BZOP: 3-(Benzoyloxy)propyl, 4BZOB: 4-(Benzoyloxy)butyl, Benzyl: Benzyl, PE: Phenethyl, S2H2P: (S)-2-Hydroxy-2-phenethyl, I3M : Indol-3-ylmethyl , 1MOI3M: 1-Methoxyindol-3-ylmethyl, 4MOI3M: 4-Methoxyindol-3-ylmethyl

Table S2 Primer sequences for RT-qPCR in this research.

Target Gene	Sequences	Primer efficiency; *P. melete* (*P. napi*)
NSP-F	AATTGGCGGCTTTATACACG	106% (91%)
NSP-R	TTCTTTCCTTCGGCACTTGT	
MA-F	TGTTGCTAACGCACTGGAAG	108% (91%)
MA-R	CCCTCCAACGCAGTAATGAT	
SDMA-F	CCACGAGCTAAGCGGTAGAG	102% (92%)
SDMA_*melete*-R	TGTCCATGGCCCTCTTAAAC	
SDMA_*napi*-R	CCATATTTCCTGCCATTCGT	
EF1α-F	AGGAATTGCGTCGTGGTTAC	104% (103%)
EF1α-R	GCAAGCAATGTGAGCTGTGT	

Table S3 Raw qPCR data of *P. melete* fed on *A. kamchatica* and *C. occulta*

Target	Plant species	Sample	Ct
primer check			
EF1α		undiluted	16.02
EF1α		1 to 10	19.09
EF1α		1 to 100	22.44
EF1α		1 to 1000	25.65
EF1α		RNA	NA
MA		undiluted	17.98
MA		1 to 10	21.07
MA		1 to 100	24.43
MA		1 to 1000	27.31
MA		RNA	30.05
NSP		undiluted	15.12
NSP		1 to 10	18.08
NSP		1 to 100	21.49
NSP		1 to 1000	24.55
NSP		RNA	34.95
SDMA		undiluted	17.2
SDMA		1 to 10	20.23
SDMA		1 to 100	23.74
SDMA		1 to 1000	26.94
SDMA		RNA	NA
1st replicate			
EF1α	*A. kamchatica*	A. kamchatica1	16.19
EF1α	*A. kamchatica*	A. kamchatica2	15.25
EF1α	*A. kamchatica*	A. kamchatica3	15.19
EF1α	*C. occulta*	C. occulta1	14.65
EF1α	*C. occulta*	C. occulta2	16.08
EF1α	*C. occulta*	C. occulta3	15.21
MA	*A. kamchatica*	A. kamchatica1	16.23
MA	*A. kamchatica*	A. kamchatica2	17.71

MA	*A. kamchatica*	A. kamchatica3	17.14
MA	*C. occulta*	C. occulta1	19.13
MA	*C. occulta*	C. occulta2	20.4
MA	*C. occulta*	C. occulta3	27.4
NSP	*A. kamchatica*	A. kamchatica1	20.21
NSP	*A. kamchatica*	A. kamchatica2	19.77
NSP	*A. kamchatica*	A. kamchatica3	20.89
NSP	*C. occulta*	C. occulta1	15.57
NSP	*C. occulta*	C. occulta2	16.14
NSP	*C. occulta*	C. occulta3	15.85
SDMA	*A. kamchatica*	A. kamchatica1	16.41
SDMA	*A. kamchatica*	A. kamchatica2	16.78
SDMA	*A. kamchatica*	A. kamchatica3	16.09
SDMA	*C. occulta*	C. occulta1	15.85
SDMA	*C. occulta*	C. occulta2	17.12
SDMA	*C. occulta*	C. occulta3	17.12
2nd replicate			
EF1α	*A. kamchatica*	A. kamchatica1	17.55
EF1α	*A. kamchatica*	A. kamchatica2	15.59
EF1α	*A. kamchatica*	A. kamchatica3	16.19
EF1α	*C. occulta*	C. occulta1	15.29
EF1α	*C. occulta*	C. occulta2	16.89
EF1α	*C. occulta*	C. occulta3	16.09
MA	*A. kamchatica*	A. kamchatica1	18.41
MA	*A. kamchatica*	A. kamchatica2	19.02
MA	*A. kamchatica*	A. kamchatica3	17.81
MA	*C. occulta*	C. occulta1	19.61
MA	*C. occulta*	C. occulta2	21.20
MA	*C. occulta*	C. occulta3	28.46
NSP	*A. kamchatica*	A. kamchatica1	21.06
NSP	*A. kamchatica*	A. kamchatica2	21.57
NSP	*A. kamchatica*	A. kamchatica3	21.83

NSP	*C. occulta*	C. occulta1	16.39
NSP	*C. occulta*	C. occulta2	17.06
NSP	*C. occulta*	C. occulta3	16.41
SDMA1	*A. kamchatica*	A. kamchatica1	18.07
SDMA1	*A. kamchatica*	A. kamchatica2	17.26
SDMA1	*A. kamchatica*	A. kamchatica3	16.93
SDMA1	*C. occulta*	C. occulta1	16.58
SDMA1	*C. occulta*	C. occulta2	17.82
SDMA1	*C. occulta*	C. occulta3	17.93

Table S4 Raw qPCR data of *P. napi* fed on four *Arabidopsis* lines

Target	Mutant	Samples	Ct
Primer check			
EF1α		undiluted	16.81
EF1α		1 to 10	18.70
EF1α		1 to 100	22.22
EF1α		1 to 1000	26.18
EF1α		RNA	NA
MA		undiluted	16.97
MA		1 to 10	20.58
MA		1 to 100	24.07
MA		1 to 1000	27.75
MA		RNA	NA
NSP		undiluted	15.47
NSP		1 to 10	19.03
NSP		1 to 100	22.49
NSP		1 to 1000	26.19
NSP		RNA	NA
SDMA		undiluted	16.21
SDMA		1 to 10	19.31
SDMA		1 to 100	23.02
SDMA		1 to 1000	26.71
SDMA		RNA	NA
EF1α	Col-0	Col0-1	19.32
EF1α	Col-0	Col0-2	18.26
EF1α	Col-0	Col0-3	19.98
EF1α	Col-0	Col0-4	18.39
EF1α	Col-0	Col0-5	19.41
EF1α	MAM1	MAM1-1	18.45
EF1α	MAM1	MAM1-2	18.05
EF1α	MAM1	MAM1-3	18.11
EF1α	MAM1	MAM1-4	17.64

EF1α	MAM1	MAM1-5	17.63
EF1α	MAM3	MAM3-1	19.08
EF1α	MAM3	MAM3-2	19.08
EF1α	MAM3	MAM3-3	19.15
EF1α	MAM3	MAM3-4	18.06
EF1α	MAM3	MAM3-5	18.74
EF1α	quadGLS	quadGLS-1	20.1
EF1α	quadGLS	quadGLS-2	18.47
EF1α	quadGLS	quadGLS-3	21.02
EF1α	quadGLS	quadGLS-4	20.02
EF1α	quadGLS	quadGLS-5	19.04
MA	Col-0	Col0-1	20.05
MA	Col-0	Col0-2	18.83
MA	Col-0	Col0-3	22.03
MA	Col-0	Col0-4	19.7
MA	Col-0	Col0-5	20.35
MA	MAM1	MAM1-1	17.98
MA	MAM1	MAM1-2	18.01
MA	MAM1	MAM1-3	17.8
MA	MAM1	MAM1-4	19.44
MA	MAM1	MAM1-5	17.26
MA	MAM3	MAM3-1	18.91
MA	MAM3	MAM3-2	18.43
MA	MAM3	MAM3-3	20.17
MA	MAM3	MAM3-4	18.26
MA	MAM3	MAM3-5	18.24
MA	quadGLS	quadGLS-1	21.04
MA	quadGLS	quadGLS-2	22.47
MA	quadGLS	quadGLS-3	22.8
MA	quadGLS	quadGLS-4	21.15
MA	quadGLS	quadGLS-5	23.09
NSP	Col-0	Col0-1	17.6

NSP	Col-0	Col0-2	17.79
NSP	Col-0	Col0-3	17.7
NSP	Col-0	Col0-4	17.48
NSP	Col-0	Col0-5	17.78
NSP	MAM1	MAM1-1	20.69
NSP	MAM1	MAM1-2	17.88
NSP	MAM1	MAM1-3	18.16
NSP	MAM1	MAM1-4	18.16
NSP	MAM1	MAM1-5	20.06
NSP	MAM3	MAM3-1	17.89
NSP	MAM3	MAM3-2	17.42
NSP	MAM3	MAM3-3	17.49
NSP	MAM3	MAM3-4	17.06
NSP	MAM3	MAM3-5	17.72
NSP	quadGLS	quadGLS-1	19.69
NSP	quadGLS	quadGLS-2	17.68
NSP	quadGLS	quadGLS-3	21.45
NSP	quadGLS	quadGLS-4	20.19
NSP	quadGLS	quadGLS-5	18.36
SDMA	Col-0	Col0-1	18.19
SDMA	Col-0	Col0-2	16.44
SDMA	Col-0	Col0-3	18.27
SDMA	Col-0	Col0-4	17.45
SDMA	Col-0	Col0-5	18.22
SDMA	MAM1	MAM1-1	17.78
SDMA	MAM1	MAM1-2	17.29
SDMA	MAM1	MAM1-3	16.22
SDMA	MAM1	MAM1-4	17.32
SDMA	MAM1	MAM1-5	17.33
SDMA	MAM3	MAM3-1	18.13
SDMA	MAM3	MAM3-2	18.57
SDMA	MAM3	MAM3-3	18.49

SDMA	MAM3	MAM3-4	17.32
SDMA	MAM3	MAM3-5	18.94
SDMA	quadGLS	quadGLS-1	19.13
SDMA	quadGLS	quadGLS-2	17.8
SDMA	quadGLS	quadGLS-3	20.67
SDMA	quadGLS	quadGLS-4	19.59
SDMA	quadGLS	quadGLS-5	18.68
